# Targeting A*β* and p-Tau Clearance in Methamphetamine-Induced Alzheimer's Disease-Like Pathology: Roles of Syntaxin 17 in Autophagic Degradation in Primary Hippocampal Neurons

**DOI:** 10.1155/2022/3344569

**Published:** 2022-05-18

**Authors:** Yuanhui Zhu, Xi Wang, Miaoyang Hu, Tingyu Yang, Huaisha Xu, Xiuwen Kang, Xufeng Chen, Lei Jiang, Rong Gao, Jun Wang

**Affiliations:** ^1^Key Lab of Modern Toxicology (NJMU), Ministry of Education; Department of Toxicology, School of Public Health, Nanjing Medical University, 818 Tianyuan East Road, Nanjing, Jiangsu 211166, China; ^2^Wujin District Center for Disease Prevention and Control, Changzhou, Jiangsu 213100, China; ^3^Department of Emergency Medicine, The First Affiliated Hospital of Nanjing Medical University, 300 Guangzhou Road, Nanjing, Jiangsu 210029, China; ^4^Department of Hygienic Analysis and Detection, Key Laboratory of Modern Toxicology, Ministry of Education, School of Public Health, Nanjing Medical University, Nanjing, China; ^5^China International Cooperation Center for Environment and Human Health, Nanjing Medical University, 818 Tianyuan East Road, Nanjing, Jiangsu 211166, China

## Abstract

Methamphetamine (Meth), a central nervous system (CNS) stimulant with strong neurotoxicity, causes progressive cognitive impairment with characterized neurodegenerative changes. However, the mechanism underlying Meth-induced pathological changes remains poorly understood. In the current study, Meth elicited a striking accumulation of the pathological proteins hyperphosphorylated tau (p-tau) and amyloid beta (A*β*) in primary hippocampal neurons, while the activation of autophagy dramatically ameliorated the high levels of these pathological proteins. Interestingly, after the Meth treatment, A*β* was massively deposited in autophagosomes, which were remarkably trapped in early endosomes. Mechanistically, syntaxin 17 (Stx17), a key soluble n-ethylmaleimide-sensitive fusion protein (NSF) attachment protein receptor (SNARE) protein responsible for autophagosome and mature endosome/lysosome fusion, was significantly downregulated and hindered in combination with autophagosomes. Notably, adenovirus overexpression of Stx17 in primary neurons facilitated autophagosome-mature endosome/lysosome fusion, which dramatically reversed the Meth-induced increases in the levels of p-tau, A*β*, beta-secretase (Bace-1), and C-terminal fragments (CTFs). Immunofluorescence assays showed that Stx17 retarded the Meth-induced A*β*, p-tau, and Bace-1 accumulation in autophagosomes and facilitated the translocation of these pathological proteins to lysosomes, which indicated the importance of Stx17 via enhanced autophagosome-mature endosome/lysosome fusion. Therefore, the current study reveals a novel mechanism involving Meth-induced high levels of pathological proteins in neurons. Targeting Stx17 may provide a novel therapeutic strategy for Meth-induced neurodegenerative changes.

## 1. Introduction

Methamphetamine (Meth), commonly known as “crystal,” is a highly addictive synthetic central nervous system (CNS) stimulant with strong neurotoxicity [1, 2]. Meth abuse is a severe public problem, and the number of abusing people is approximately 37 million worldwide since 2017 [3]. It can directly penetrate the blood-brain barrier and increase of the risk to develop irreversible brain damage and multiple neurodegenerative diseases such as Alzheimer's disease (AD) and Parkinson's disease (PD) [4, 5]. To date, most studies on Meth-induced neuronal damage are available on the issue of cell apoptosis/death and pathological changes; however, most of these pathological damages are irreversible in neurons [6, 7]; therefore, some early events and interventions for Meth-induced pathological damage are required.

Neurons are cells that highly depend on intracellular degradation pathways to clear pathological proteins, where autophagy plays a critical role. Autophagy is a crucial cellular degradative process for defective organelle and pathological protein clearance over a neuron's lifetime [8]. In the autophagic process, the isolated membrane expands and forms the double-membrane autophagosome to package the cytoplasmic component and delivers it to the late endosome to form amphisomes, which fuses with lysosomes for degradation [9]. Autophagosomes can also fuse with lysosomes to form autolysosomes, which act as the terminal processor for pathological protein degradation. Epidemiological studies have demonstrated that mutations in autophagy-related genes lead to familial Alzheimer's disease (FAD) [10]. Furthermore, the autophagic markers microtubule-associated protein 1 light (LC3) and p62 obviously accumulate in AD patients, which suggests defects in autophagic flux [11]. Recently, one study revealed an aberrant expression of LC3 and ATG5 accompanied by characterized neurodegenerative changes in the prefrontal cortex of postmortem Meth users [12]. Similarly, an in vivo study suggested that administration of Meth enhances the autophagy-associated mRNA levels, including Atg2a, Atg5, Atg14, and Atg16L1 in the rat dorsal striatum; meanwhile, the levels of p53 and caspase-9 increased [12]. To strengthen the Meth-induced AD-like changes, our previous works showed that Meth exposure greatly upregulated amyloid beta (A*β*) and hyperphosphorylated tau (p-tau) [13–15], which are two principle biomarkers for AD-like pathological changes. Most recently, an in vitro study also revealed defects in autophagic flux mediated by Meth in primary neurons in our work [16]. Nonetheless, the association between autophagy and Meth-induced pathological protein accumulation in neurons, including A*β* and p-tau, remains ambiguous.

As the rate-limiting step in the autophagy process, autophagosome-lysosome fusion plays key roles in AD-like pathological protein degradation [17, 18]. Syntaxin17 (Stx17), a protein in the syntaxin family with a unique C-terminal hairpin structure, is a key player in the soluble NSF attachment protein receptor (SNARE) complex. Interestingly, Stx17 is located only in the completed autophagosome membrane to bind the LC3-interacting region (LIR) and mature (late) endosome, and defects in Stx17 may resist the fusion process to form incomplete autophagosomes [19], which retards the autophagosome-late endosome/lysosome fusion.

The endosome is an organelle originating from the trans-Golgi network that participates in endocytic membrane transport. It commonly serves as the main organelle to package pathological proteins into lysosomes for degradation [20]. According to the stage of endosomal recycling pathways, endosomes can be classified by early, recycling, and late endosomes [21]. Early endosomes drive from the endocytic pathway to package pathological proteins outside the cells, sort their packaged proteins, and acidify them by acid hydrolases to form late endosomes. Late endosomes can directly fuse with autophagosomes to form “amphisomes” with packaged proteins within cells [22]. Therefore, in the current study, we investigated the pathological proteins A*β* and p-tau, which exist as cargos in autophagosomes and endosomes mediated by Meth. Moreover, the pivotal roles of Stx17 in ameliorating the Meth-induced pathological protein accumulation, which is linked to the autophagosome-late endosome/lysosome fusion, were discussed.

## 2. Materials and Methods

### 2.1. Chemicals

Meth was obtained from the National Institutes for Food and Drug Control (Beijing, China). Rapamycin (Rapa, AY 22989) was purchased from LC Laboratories (Woburn, MA, USA). 3-Methyladenine (3-MA, A8353) was purchased from APExBIO Technology LLC (Houston, TX, USA). Bafilomycin A1 (Baf A1, B1793) was obtained from Sigma-Aldrich (St. Louis, MO, USA), FAM-A*β*_1-42_ (green fluorescence) was purchased from AnaSpec Manufactures peptides (Fremont, CA, USA), and mRFP-GFP-LC3 was purchased from AnaSpec Manufactures peptides (Fremont, CA, USA).

### 2.2. Primary Neuron and Cell Culture and Treatment

Primary E18 rat hippocampal neurons were isolated and cultured in neurobasal medium (Invitrogen, 21003-049) supplemented with 2% B-27 serum-free supplement (Invitrogen, 17504-044), 0.5% penicillin/streptomycin (Multicell Techs Inc., 450-201-EL), and 0.1% L-glutamine (Gibco, 25030-081) for 10-12 days. The numbers of MAP-2-positive cells (>95%) were detected by immunofluorescence to identify the purity of the hippocampal neurons. Neurons were pretreated with Rapa, 3-MA, or Baf A1 for 1 h; then, Meth was added and incubated for 24 h. BV2 microglial cells were obtained from the American Type Culture Collection (ATCC, Manassas, VA, USA) and cultured in Minimum Essential Medium (MEM; HyClone, Logan, UT, USA) supplemented with 10% fetal bovine serum (FBS; Gibco, Grand Island, NY, USA), 100 U/ml penicillin, and 0.1 mg/ml streptomycin. BV2 cells were incubated with Meth (900 *μ*m) for 24 h and then subjected to FAM-A*β*_1-42_ (1 *μ*m/L) for 3 h. All the experiments were approved by the Animal Experimental Ethics Committee of Nanjing Medical University (permission number: IACUC-1902013).

### 2.3. Adenoviral Vector Transfection

Adenoviral vectors carrying 3 × flag-Stx17 (HB-AP2100001) and an adenoviral vector carrying mRFP-GFP-LC3 (HB-AP2100001) were purchased from Hanbio Biotechnology (Shanghai, China), and transfection was performed according to the manufacturer's instruction manual. The cells were incubated with the indicated reagents for further experiments after 24 h transfection.

### 2.4. Enzyme-Linked Immunosorbent Assay (ELISA)

Cell culture medium was collected, and A*β*_1-42_ secretion by neurons was detected. The levels of A*β*_1-42_ were determined by ELISA kits (Elabscience, E-EL-R1402c) according to the manufacturer's instructions. All ELISA experiments were performed in triplicate and repeated at least three times [23].

### 2.5. Immunoblotting

Total proteins were extracted from rat hippocampal neurons in ice-cold RIPA buffer (Sigma–Aldrich, #SLCD5849) containing phosphatase inhibitor cocktails (1 : 500, Sigma–Aldrich, 41659200) and protease inhibitor. Proteins were concentrated by a BCA protein assay (Thermo Fisher Scientific, 23227). The proteins were separated by electrophoresis on a 12.5% SDS–PAGE gel (Epizyme Biotechnology, PG113) and transferred to polyvinylidene difluoride membranes (Millipore Corporation, IPVH00010), which were blocked with 3% bovine serum albumin for 1 h at room temperature. Then, the membranes were incubated at 4 °C overnight with the primary antibody in Tris-buffered saline containing 0.1% Tween and 3% bovine serum albumin. The following primary antibodies and fluorescent reagents were used: the anti-LAMP1 (1:100, ab62562), anti-LAMP1 (1:100, ab25630), anti-SQSTM1/p62 (1:100, ab109012), anti-Stx17 (1:100, ab229646), anti-Amyloid 1-42 (1:100, ab10148), and anti-tau phosphor S396 (1: 200, ab109390); the antibodies were purchased from Abcam. The anti-LC3 (1:100, 3868S), anti-Rab5 (1:100, 3547 T), and anti-Rab7 (1:100, 9367S) antibodies were obtained from Cell Signaling Technology. The anti-LC3 (1:50, sc-271625) and anti-Bace-1 (1:50, sc-33711) antibodies were from Santa Cruz Biotechnology, and the anti-Bace-1 antibody was from Proteintech (1:50,12807-1-AP). The membranes were washed for 3 times and incubated at room temperature with Goat Anti-Rabbit IgG (H + L) or Goat Anti-Mouse IgG (H + L) (1 : 20000, Biosharp, sc-2004 and sc-2005, respectively). Western blotting results were analyzed by ImageJ software to measure the intensities of the bands. The results were normalized to the housekeeping gene *β*-actin, and each experiment was repeated more than 3 times.

### 2.6. Immunofluorescence and Image Acquisition

The neurons were washed with PBS (pH = 7.4) (Boster, AR0030) and fixed with methyl alcohol (Sinopharm Chemical Reagent, 30188928) for 10 min at -20 °C. Then, the cells were permeabilized with 0.3% Triton X-100 (Sinopharm Chemical Reagent, 20180830) and blocked with 10% goat serum (Boster, AR0009) for 1 h. Afterwards, the cells were incubated with primary antibodies overnight at 4 °C; then, the fluorescent secondary antibodies were incubated for 2 h, followed by DAPI (Abcam, ab104139) staining for 15 min. The stained cells were visualized using a Zeiss LSM710 confocal microscope. The following primary antibodies and fluorescent reagents were used: anti-LC3 (Cell Signaling Technology, 3868S), anti-LC3 (Santa Cruz Biotechnology, sc-271625), anti-LAMP1 (Abcam, ab56416), anti-LAMP1 (Abcam, ab25630), anti-Rab5 (Cell Signaling Technology, 3547 T), anti-Rab7 (Cell Signaling Technology, 9367S), anti-SQSTM1/p62 (Abcam, ab109012), anti-Stx17 (Abcam, ab229646), anti-Bace (Proteintech, 12807-1-AP), anti-Bace (Santa Cruz Biotechnology, sc-33711), anti-Amyloid 1-42 (Abcam, ab10148), anti-tau phosphor S396 (1 : 5000, Abcam, ab109390), SignalBoost Immunoreaction Enhancer Kit (Merck, #407207), Alexa Fluor 488-conjugated goat anti-mouse IgG (H + L) (Invitrogen, A-11029), Alexa Fluor 488-conjugated goat anti-rabbit IgG (H + L) (Invitrogen, A-11008), Alexa Fluor 568-conjugated goat anti-rabbit IgG (H + L) (Invitrogen, A-11036), Alexa Fluor 488-affinipure goat anti-rabbit IgG (H + L) (Yeasen, A27160), and Alexa Fluor 594-affinipure goat anti-rabbit IgG (H + L) (Yeasen, A27630). Cells in nine random fields for each dish were counted, with three repeated times. The immunolabeling intensity of the antibodies was quantified from the original confocal images taken with the same lens under identical conditions with identical settings for laser gains and photomultiplier tube gains. Imaging series were analyzed by Image J (National Institutes of Health).

### 2.7. Statistical Analysis

Statistical analysis was performed using the SPSS software (Version 25.0). All experimental statistics are expressed as the means ± SEMs. Statistical significance of the *p* value was calculated by one-way ANOVA and Dunnett's multiple comparison procedures. All tests of statistical significance were two-sided, and *p* < 0.05 was considered significant.

## 3. Results

### 3.1. Meth Exposure Leads to Autophagic and AD-Like Pathological Protein Accumulation in the Primary Hippocampal Neurons

**Figure 1:**
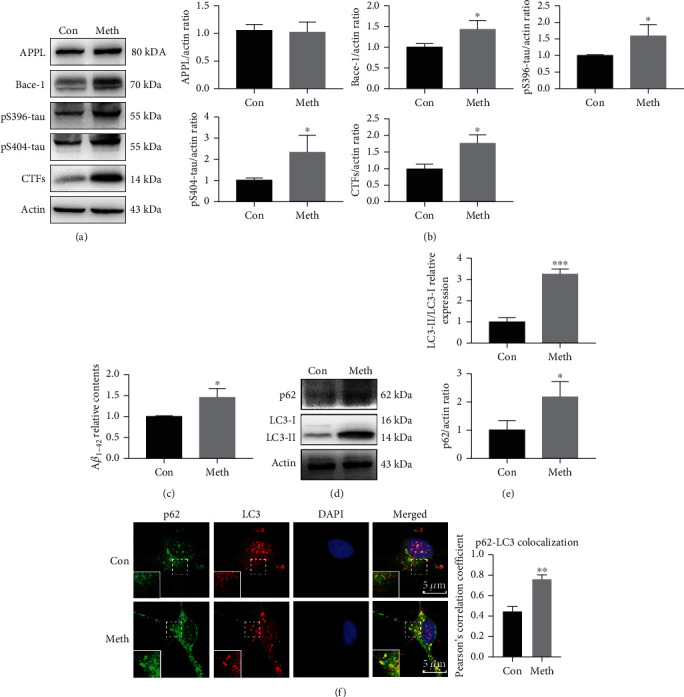
Meth treatment increased autophagy and AD-like pathological protein expression. (a) Western blot analysis of APPL, Bace-1, pS396-tau, pS404-tau, and CTFs in primary hippocampal neurons. (b) *β*-actin levels were assessed and compared with the control group. (c) ELISA analysis of A*β*_1-42_ levels after Meth exposure. (d) Autophagy markers LC3 and p62 were detected by western blot. (e) LC3-II/LC3-I protein expression levels were assessed and served as the controls, while p62/*β*-actin levels were assessed and compared with the control group (∗*p* < 0.05 and ∗∗∗*p* < 0.001 compared with the control group). (f) Co-localization of LC3 and p62 by an immunofluorescence analysis. Co-localization of LC3 and p62 was quantified and expressed as Pearson coefficient value (∗∗*p* < 0.01 compared with the control group). Scale bar: 5 *μ*m.

Primary hippocampal neurons were incubated with 900 *μ*m Meth for 24 h, after which AD-like pathological proteins were detected by western blot and ELISA. The concentration was chosen based on the Meth concentrations found in the blood, urine, and tissue of individuals who abuse Meth [24, 25]. It showed that the AD-associated pathological proteins, including Bace-1, CTFs, pS396-tau, and pS404-tau, were significantly upregulated after Meth exposure (Figures 1(a) and 1(b)). In parallel, the excretion level of A*β*_1-42_ was notably increased by the ELISA assay (Figure 1(c)), which suggested that Meth promotes AD-like pathological proteins accumulation.

Since AD-like pathological protein changes are closely associated with the autophagic pathway [26–28]; therefore, the autophagic activities after Meth treatment were examined. LC3 and the autophagic substrate p62, which are two principle autophagic markers, were detected; meanwhile, the co-localization of LC3 and p62 was examined after the Meth treatment. Meth elicited a pronounced conversion of LC3-I to LC3-II, a significant elevation of the LC3-II/LC3-I ratio, and a marked increase expression of p62 (Figures 1(d) and 1(e)). Notably, the co-localization (yellow puncta) of LC3 (marked in red) and p62 (marked in green) was remarkably increased in Meth-treated neurons compared with control neurons (Figure 1(f)), which implies defects in the autophagic flux.

### 3.2. Autophagy Activation Attenuates Meth-Induced AD-Like Pathological Protein Expression

**Figure 2:**
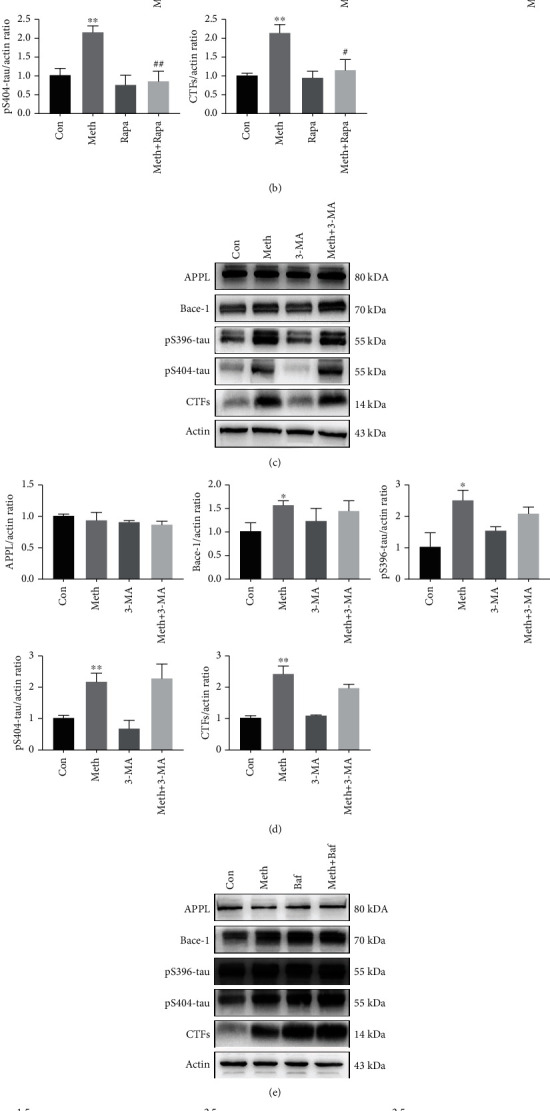
Effects of autophagy on Meth-induced AD-like pathological protein changes. (a) Western blot analysis of the effect of Meth (900 *μ*m) and Rapa (25 *μ*m) on APPL, Bace-1, pS396-tau, pS404-tau, and CTFs in primary hippocampal neurons. (c) Western blot analysis of the effect of Meth (900 *μ*m) and 3-MA (10 mM) on APPL, Bace-1, pS396-tau, pS404-tau, and CTFs in primary hippocampal neurons. (e) Western blot analysis of the effect of Meth (900 *μ*m) and Baf (20 nM) on APPL, Bace-1, pS396-tau, pS404-tau, and CTFs in primary hippocampal neurons. (b, d, f) *β*-actin levels were assessed and compared with the control group (∗*p* < 0.05 and ∗∗*p* < 0.01 compared with the control group; #*p* < 0.05 and ##*p* < 0.01 compared to the Meth group).

To explore whether Meth-induced AD-like pathological protein accumulation was attributed to abnormal autophagy, Rapa, a specific autophagy agonist, 3-MA, an inhibitor of autophagy, and Baf, a blocker of lysosomes, were used. As expected, the protein levels of Bace-1, CTFs, pS396-tau, and pS404-tau significantly increased after the Meth treatment; an effect was ameliorated when cells were pretreated with Rapa (Figures 2(a) and 2(b)). To strengthen the effects of Rapa on the decrease of phospho-tau and CTFs levels, the autophagic flux was detected by the double-labeled adenovirus mRFP-GFP-LC3. When autophagosomes fuse with lysosomes, the acid-sensitive green GFP fluorescence is quenched, and residual red fluorescence is observed. In Figure S1, the ratio of yellow puncta dramatically increased in Meth group, an effect was ameliorated by Rapa treatment (Figures S1A and S1C). The similar results verified by the western blot that Rapa treatment ameliorated the Meth-induced upregulation of LC3-IIand p62 (Figures S1B and S1D). However, the autophagy inhibitor 3-MA exhibited no significant changes in Meth-induced AD-like pathological protein expression (Figures 2(c) and 2(d)). In addition, pretreatment with Baf accelerated the Meth-induced pS396-tau, pS404-tau, and CTFs expression, and this treatment did not significantly alter the expression of Bace-1 protein (Figures 2(e) and 2(f)). These results collectively indicate the pivotal roles of autophagy in the clearance of Meth-induced pathological protein degradation.

### 3.3. Meth Exposure Facilitates Endosome Accumulation and Stx17 Downregulation

**Figure 3:**
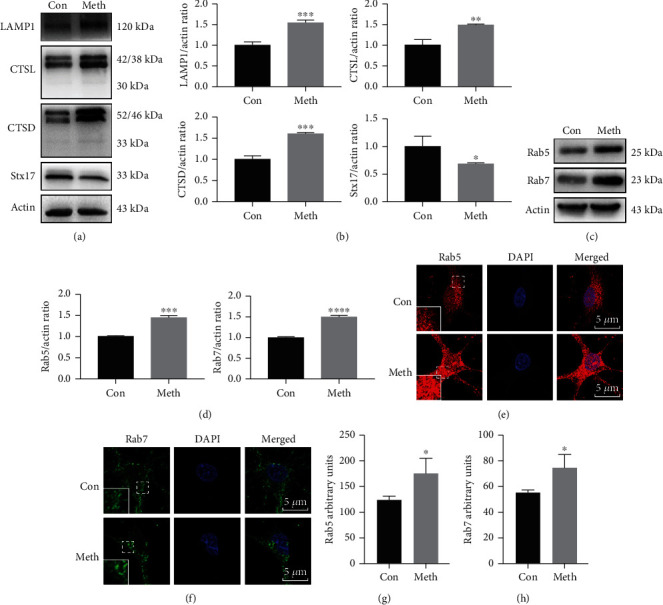
Effect of Meth on LAMP1, CTSL, CTSD, Stx17, Rab5, and Rab7 expression. (a) Western blot analysis of the LAMP1, CTSL, CTSD, and Stx17 expression in primary hippocampal neurons. (c) Western blot analysis of the Rab5 and Rab7 expression in primary hippocampal neurons. (b, d) *β*-actin levels were assessed and compared with the control group (∗*p* < 0.05, ∗∗*p* < 0.01, ∗∗∗*p* < 0.001, and ∗∗∗∗*p* < 0.0001 compared with the control group). (e) Immunofluorescence analysis of Rab5 expression. (f) Immunofluorescence analysis of Rab7 expression. Scale bar: 5 *μ*m. (g, h) Quantification of the mean fluorescence intensity (MFI) in the confocal images showed the Rab5 and Rab7 levels (∗*p* < 0.05 compared with the control group).

Since lysosomal proteolytic enzymes are crucial in the degradation of cargo in the autophagic process [29], we detected the expression of LAMP1, CTSL, and CTSD. Surprisingly, the expression level of LAMP1 increased after the Meth challenge, while the proteases CTSL and CTSD were markedly upregulated (Figures 3(a) and 3(b)), which implies that the lysosome and its proteolytic activity may not directly impact the Meth-induced upregulation of pathological proteins. To unravel the contradictory phenomenon between lysosome activation and pathological protein accumulation, we hypothesized that membrane fusion defects may occur between autophagosomes and mature endosomes/lysosomes. Therefore, Stx17, an important SNARE protein that responds to autophagosome and mature endosome/lysosome fusion, was examined. As expected, the Stx17 expression was strikingly reduced after Meth exposure. In addition, endosomes, which play a key role in autophagosome-lysosome fusion [30], were assessed. As shown in Figure 3(a), Stx17 was significantly downregulated after the Meth treatment, while Rab5 is a marker of early endosome, and Rab7, an indicator of late endosome, increased (Figures 3(c) and 3(d)). To further confirm the western blot results, the immunofluorescence results showed a marked increase in Rab5 fluorescence intensity and the accumulated Rab7 GFP puncta (Figures 3(e)–3(h)) challenged with Meth.

### 3.4. Stx17 Reverses the Meth-Induced Autophagosome-Late Endosome/Lysosome Fusion Deficiency

**Figure 4:**
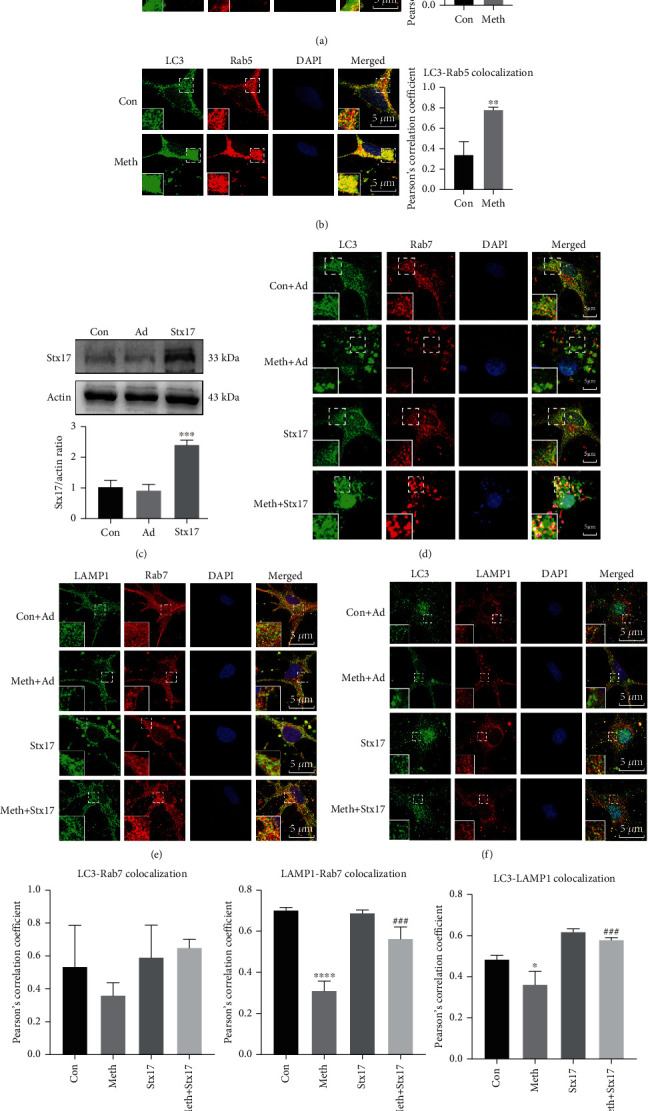
Stx17 overexpression recovered the Meth-induced autophagosome-late endosome/lysosome fusion deficiency. (a) Co-localization of LC3 and Stx17. (b) Co-localization of LC3 and Rab5. (c) pHBAd-EF1-Stx17-3 flag (MOI = 40, 36 h) or empty adenovirus vector (Ad) was overexpressed in primary hippocampal neurons. (d) Co-localization of LC3 and Rab7 after the Stx17 overexpression. (e) Co-localization of LAMP1 and Rab7 after the Stx17 overexpression. (f) Co-localization of LC3 and LAMP1 after the Stx17 overexpression. Scale bar: 5 *μ*m. (g) Co-localization of LC3-Rab7, LAMP1-Rab7, and LC3-LAMP1 was quantified and expressed as Pearson coefficient value (∗∗*p* < 0.01 and ∗∗∗*p* < 0.001 compared with the control group and ###*p* < 0.001 compared to the Meth group).

As an SNARE family member, Stx17 is located in the endoplasmic reticulum (ER) under physiological conditions and normally relocates to mature autophagosomes [31]. As depicted in Figure 4, Meth elicited massive accumulation of autophagosomes, which manifested as the deposition of LC3 puncta. Interestingly, most of the Stx17 was located around instead of co-localizing with the LC3 puncta (Figure 4(a)), demonstrating the deposition of the immature autophagosomes. To explore the spatial distribution of endosomes and autophagosomes after Meth treatment, LC3, Rab5, and Rab7 were labeled by fluorescent staining. LC3 and Rab5 co-localization was dramatically enhanced after Meth exposure. In contrast, the overlap between LC3 and Rab7 decreased (Figures 4(b) and 4(d)). To determine whether the autophagy-late endosome/lysosome fusion defect was linked to Stx17, the recombinant adenoviruses were used to overexpress Stx17 in primary hippocampal neurons (Figure 4(c)). Comparing to that in the Meth-treated group, the overlap between LC3 and Rab7 increased in the Meth+Stx17 group (Figures 4(d) and 4(g)). Moreover, the co-localization of Rab7 and LAMP1, LC3, and LAMP1 was substantially enhanced after the Stx17 overexpression compared with the Meth-treated group (Figures 4(e)–4(g)). Taken together, these results imply a key role of Stx17 downregulation in Meth-induced autophagosome-late endosome/lysosome fusion deficiency, since the Stx17 overexpression facilitates the autophagosome-late endosome/lysosome fusion, which improves autophagic flux.

### 3.5. Stx17 Attenuates Meth-Induced Pathological Protein Expression

**Figure 5:**
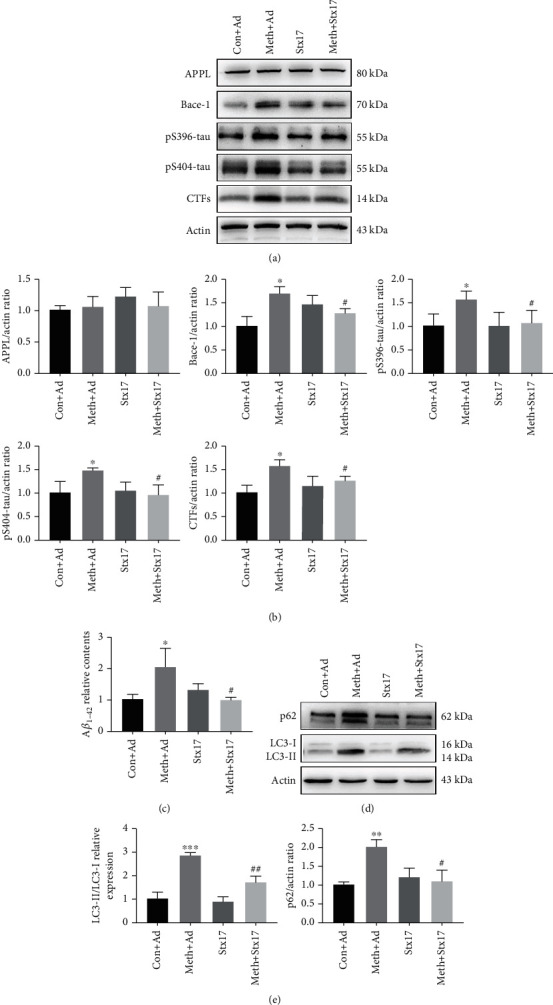
AD-like pathological protein and autophagy markers expression changes after the Stx17 overexpression. (a) Western blot analysis of APPL, Bace-1, pS396-tau, pS404-tau, and CTFs in primary hippocampal neurons after Stx17 overexpression. (b) *β*-actin levels were assessed and compared with the control group. (c) ELISA analysis of A*β*_1-42_ levels after the Stx17 overexpression. (d) Western blot analysis of LC3 and p62 in primary hippocampal neurons after the Stx17 overexpression. (e) *β*-actin levels were assessed and compared with the control group (∗*p* < 0.05, ∗∗*p* < 0.01, and ∗∗∗*p* < 0.001 compared with the control group; #*p* < 0.05 and ##*p* < 0.01 compared to the Meth group).

Since Meth exposure contributes to the aforementioned deficiency of the autophagosome-late endosome/lysosome fusion, we sought to examine whether the defects affected the pathological protein expression. Therefore, western blot and ELISA were applied to detect the protein expression of AD-like pathological proteins and autophagic markers. It showed that pathological proteins, including Bace-1, CTFs, A*β*_1-42_, pS396-tau, and pS404-tau, were significantly increased after the Meth exposure, and these effects could be substantially reversed by Stx17 overexpression (Figures 5(a)–5(c)). Additionally, Stx17 overexpression effectively attenuated the Meth-induced LC3 and p62 upregulation (Figures 5(d) and 5(e)), which suggested that the improvement of the autophagosome-late endosome/lysosome fusion by the Stx17 overexpression helps to clear the pathological proteins.

### 3.6. Meth Promotes the Deposition of Pathological Proteins in Autophagosomes, While Stx17 Overexpression Facilitates the Pathological Protein Degradation in Lysosomes

**Figure 6:**
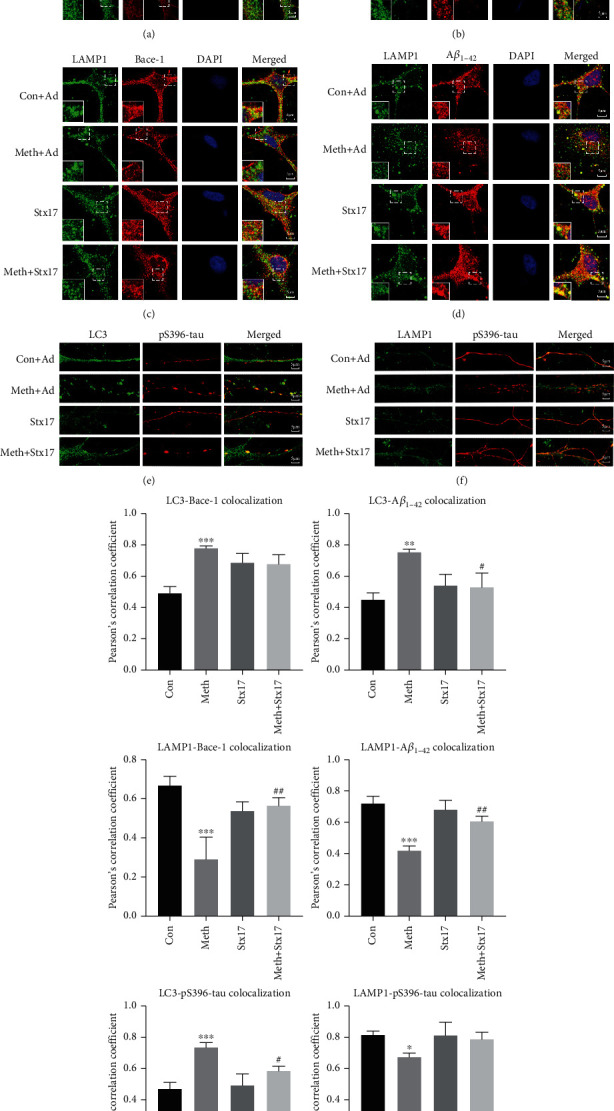
Co-localization changes of LC3, LAMP1, and AD-like pathological proteins after the Stx17 overexpression. (a) Co-localization of LC3 and Bace-1 after the Stx17 overexpression. (b) Co-localization of LC3 and A*β*_1-42_ after the Stx17 overexpression. (c) Co-localization of LAMP1 and Bace-1 after the Stx17 overexpression. (d) Co-localization of LAMP1 and A*β*_1-42_ after the Stx17 overexpression. (e) Co-localization of LC3 and p-S396tau after the Stx17 overexpression. (f) Co-localization of LAMP1 and pS396-tau after the Stx17 overexpression. Scale bar: 5 *μ*m.(g) Co-localization of LC3-Bace-1, LC3-A*β*_1-42_, LAMP1-Bace-1, LAMP1-A*β*_1-42_, LC3-pS396-tau, and LAMP1-pS396-tau was quantified and expressed as Pearson coefficient value (∗*p* < 0.05,∗∗*p* < 0.01, and ∗∗∗*p* < 0.001 compared with the control group; #*p* < 0.05 and ##*p* < 0.01 compared to the Meth group).

Since Stx17 overexpression substantially relieved the aforementioned pathological protein accumulation, it was logical to investigate the potential mechanisms of the Stx17-mediated pathological protein clearance. We first explored the spatial localizations of pathological proteins (Bace-1, A*β*_1-42_, and p-S396 tau), autophagosomes, and lysosomes. As depicted in Figures 6(a) and 6(b), pathological proteins including Bace-1 and A*β*_1-42_ were accumulated and restrained in cytoplasmic autophagosomes after Meth treatment, while p-tau, which trapped in autophagosomes, was mainly accumulated in axons (Figures 6(e)–6(g)). Notably, Stx17 overexpression significantly retarded the Meth-mediated trapping of Bace-1, A*β*_1-42_, and p-tau in autophagosomes and exhibited less co-localization of Bace-1, A*β*_1-42_, and p-tau with LC3. In Meth-treated neurons, the scarce co-staining of Bace-1, A*β*_1-42_, and p-tau with LAMP1 was observed, while Stx17 overexpression drove Bace-1 and A*β*_1-42_ in lysosomes (Figures 6(c) and 6(d)), since more co-staining of Bace-1 and A*β*_1-42_ with LAMP1 was shown (yellow puncta). Furthermore, Stx17 overexpression facilitated p-tau and LAMP1 co-localization in axons. These results unravel the mechanisms involving the Stx17-mediated clearance of Bace-1, A*β*_1-42_, and p-tau (Figures 6(e) and 6(f)).

### 3.7. Meth Impedes A*β*_1-42_ Clearance in Microglial Cells

It is well known the importance of the microglial cells in the pathological AD proteins' clearance [32]; we examined whether Meth exposure affects this process. As shown in Figures S2A and S2B, compared with the control group, a significant accumulation of A*β*_1-42_ was observed in Meth treated cells; meanwhile, the co-localization of lysotracker and A*β*_1-42_ decreased though the number of lysosomes increased in the Meth group, implying the defects of A*β*_1-42_ clearance. To unravel this phenomenon, the co-localization of autophagosome and A*β*_1-42_ was investigated. As expected, A*β*_1-42_ was overlapped with LC3, indicating the deposition of A*β*_1-42_ in the autophagosomes (Figures S2A and S2B). These results suggest that, similar with the phenomenon in neurons, Meth treatment decreases the ability of pathological AD proteins' clearance in microglial cells.

## 4. Discussion

Our previous studies have shown a significant increase in AD-like pathological protein expression mediated by Meth, including A*β* and p-tau [15]. These effects and mechanisms were focused on A*β* and p-tau generation. Since the balance of generation and degradation determines the pathological protein accumulation, the degradative way especially through autophagy that was investigated in the current study.

In neurons, A*β* is mainly located in the soma, while p-tau is located in the axon. A*β* and p-tau are mainly degraded through the autophagy-lysosome pathway [33]. In the process of vesicle trafficking, cargos are mainly transported by autophagic vesicles that fuse with mature endosomes or lysosomes for degradation [30]. As potential cargos, A*β* and p-tau can be delivered to lysosomes for degradation [33]. In the present work, Meth upregulated the levels of A*β* and p-tau, and as expected, the activation of autophagy by Rapa substantially impeded the enhancement of these pathological proteins mediated by Meth, suggesting that the pharmacological induction of autophagy by Rapa decreases the intracellular A*β* and p-tau levels and reduces the A*β* plaque load and the expression of tau tangles [34, 35]. Recently, two kinds of LC3 particles were observed at with different locations: vacuoles LC3 particles and free cytosolic LC3 particles. With Rapa treatment, the compartmentalization of LC3 particles within vacuoles was dramatically enhanced, while under the Meth treatment, the compartmentalization of LC3 particles within vacuoles was lost, though the total LC3 level was obviously increased [36]. To unravel the effects of Rapa on the decrease of phospho-tau and CTFs levels, the autophagic flux was detected by the double-labeled adenovirus mRFP-GFP-LC3. It showed that Rapa treatment substantially rescues the Meth-induced autophagic flux defects and the similar results were verified by the protein level that; Rapa treatment ameliorated the Meth-induced upregulation of LC3-IIand p62 (Figure S1). These evidence suggest that Rapa rescues the defects of autophagy induced by Meth in neurons. Therefore, the enhanced clearance of A*β* and p-tau may be ascribed to the restore of autophagic flux [37], which confirms the critical roles of autophagy in A*β* and p-tau clearance. Noteworthily, it is known the importance of the inflammatory cells on the pathological AD proteins' clearance, especially the microglia [32]. In the present study, a significant reduction of pathological AD proteins' clearance was also observed in microglial cells, highlighting that Meth treatment not only stimulated the accumulation of A*β*_1-42_ in neurons, but also decreased the ability of microglia for pathological AD proteins' clearance.

The mechanisms that involve the disturbance of the autophagic flux are complex. Lysosome and its proteolytic enzymes play critical roles in clearing aging-damaged organelles and degrading pathological proteins in the autophagic degradation pathway. Intriguingly, in the present study, the lysosomal marker protein LAMP1 and the lysosomal proteolytic enzyme CTSD and CTSL were significantly increased after Meth exposure, implying that the enhanced proteolytic effects was not participated in Meth-induced p-tau, Bace-1, and CTFs expression. Based on the results of the disturbance of the autophagic flux depicted in Figure S1, the disturbance of autophagosome-lysosome fusion deciphering Meth-induced pathological protein upregulation, which was in line with the results that Rapa, one agonist for promoting autophagic flux, significantly attenuated Meth-induced enhancement of the pathological proteins. Therefore, Stx17, one pivotal protein responsible for autophagosome-lysosome fusion [16], was investigated in the present work. However, other mechanisms may also be involved in, since the accumulation of A*β* causes the endosome enlargement; these enlarged endosomes fail to fuse with lysosomes for degradation in neurons [38]. We suspect that a positive feedback pathway of A*β*-autophagic flux disorder may participate in this process; therefore, more studies in this field are warranted.

The accumulation of vesicles is one of the most characterized properties inside cells and contributes to neuronal damage [39], and a similar phenomenon was observed in Meth-treated neurons. Here, early endosomes, mature endosomes, and autophagosomes were increased. To decipher the distribution of the pathological proteins, the association among these vesicles was examined. Intriguingly, Meth treatment elicited a marked co-localization of autophagosomes and early endosomes; in contrast, overlap between autophagosomes and mature endosomes was scarce. Studies have indicated that instead of early endosomes, mature endosomes can directly fuse with lysosomes [40]; in the current study, less co-localization of Rab7 and LC3, LC3 and LAMP1, and Rab7 and LAMP1 was observed, validating the Meth-induced deficiency in autophagic flux.

As the key tethering factor in the fusion process, Stx17 is derived from the cytoplasm, endoplasmic reticulum (ER), and mitochondria and plays an important role in autophagosome-late endosome/lysosome fusion [31]. In normal growing cells, Stx17 is located in the ER, mitochondria, and cytoplasm. During the autophagosome formation, the isolation membranes elongate and combine with Stx17 to form complete autophagosomes [41], whereas the impairment of autophagy may prevent Stx17 translocation to autophagosomes and form incomplete autophagosomes [41–43]. Here, Meth treatment significantly decreased the Stx17 expression, induced enlarged LC3-positive puncta accumulation, and hindered the binding of Stx17 to autophagosomes, which implied that loss of Stx17 increases immature autophagosomes under the Meth challenge. Therefore, the acquisition of Stx17 represents a pivotal step for autophagosomal fusion with lysosomes. In the present work, the Stx17 overexpression enhanced the fusion of autophagosome-mature endosomes/lysosomes, which confirmed the pivotal step of Stx17 in mediating the fusion process [15, 44].

In AD, abnormal enlargement of Rab5^+^ early endosomes and LC3^+^ autophagosomes is observed in individuals with sporadic AD, and these abnormalities precede both the onset of dementia and the emergence of plaques and tangles [45]. Therefore, the distribution of AD-like pathological proteins was assessed. As expected, Bace-1 and A*β*_1-42_ were engulfed by autophagosomes after the Meth challenge; however, these pathological proteins co-localized in immature autophagosomes and could not be degraded.

With the Stx17 overexpression, the accumulation of A*β*_1-42_ and Bace-1 inside autophagosomes substantially decreased, while the delivery of A*β*_1-42_ and Bace-1 to lysosomes was enhanced. This evidence reveals the Meth-mediated AD-like pathological protein accumulation and the pivotal roles of Stx17 in pathological protein clearance. Nonetheless, the exact roles of Stx17 in clearing pathological proteins remain ambiguous. Recent studies have clarified that the pathological proteins stored in Rab5 transport to Rab7 via the promotion of Rab5-to-Rab7 conversion. In the case of endosome loading, early endosome Rab5 recruits Mon1-Ccz1; then, Mon1-Ccz1 recruits and activates late endosome Rab7 to replace Rab5 [40, 46, 47]. One hypothesis is that Stx17 may promote the endosomal conversion from early endosomes to late endosomes, facilitate the amphisome formation, and finally fuse with lysosomes; however, the potential mechanisms needed to be validated. In addition, blocking fusion with Stx17 knockdown reduces the recruitment of dynein motors to autophagosomes, which decreases the fusion with lysosomes and finalizes the autophagosome accumulation [48]. Therefore, the link in the Stx17-dynein signaling axis cannot be ignored and deserves further investigation in future work.

**Figure 7:**
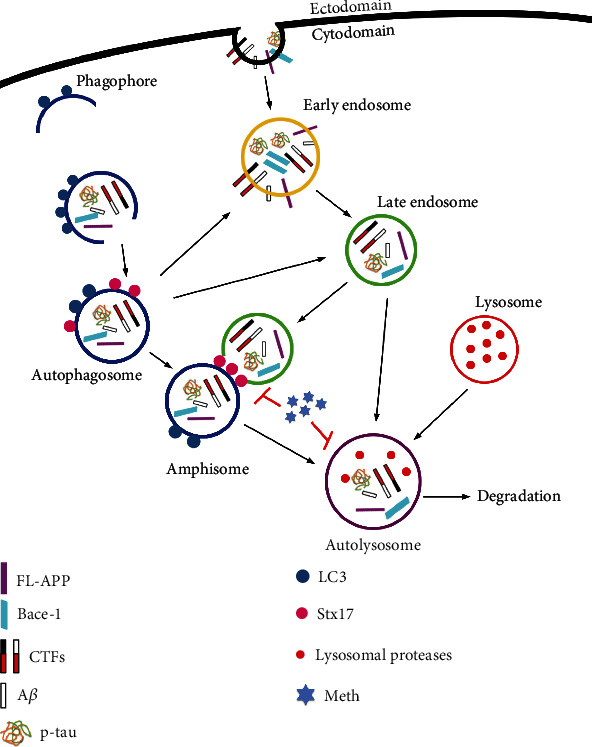
A schematic depicting the mechanism of autophagosome-late endosome/lysosome fusion deficiency in Meth-induced AD-like pathological protein accumulation. Under normal conditions, the AD-like pathological proteins (A*β*, p-tau, Bace-1, and CTFs) are engulfed by early endosomes and autophagosomes, which then transport and fuse with the late endosomes/lysosomes for degradation. Meth exposure retains the pathological proteins in autophagosomes trapped in early endosomes, blocks the fusion of autophagosomes and late endosomes/lysosomes, contributes to the pathological protein accumulation, and finalizes the neuronal damage.

Collectively, the current study reveals a novel mechanism of Meth-induced pathological protein accumulation due to autophagic flux disorder. This progress may be particularly ascribed to autophagosome-late endosome/lysosome fusion impairment. Notably, Stx17 plays key roles in the fusion process, whereas Meth treatment downregulates its expression, resists the degradation of pathological proteins, and finalizes AD-like changes (Figure 7).

## Data Availability

Data are available on request to the authors.

## Supplementary Materials

Supplementary Materials Figure S1. Rapa rescues autophagosome–lysosome fusion deficiency induced by Meth in neurons. (a, c) Hippocampal neurons were transiently transfected with an RFP-GFP tandem fluorescent-tagged LC3 (RFP-GFP-LC3) and indicated with rapa and Meth (900 *μ*m) for 24 h. The formation of autophagosome (RFP-positive; GFP-positive) and autophagolysosome (RFP-positive; GFP-negative) were detected and quantified by ImageJ (∗∗∗∗*p* < 0.0001 compared with the control group, ###*p* < 0.001 compared to the Meth group). Scale bars: 5 *μ*m. (b, d) Western blot analysis of the effect of Meth (900 *μ*m) and Rapa (25 *μm*) on LC3 and p62 in primary hippocampal neurons. LC3-II/LC3-I protein expression levels were assessed and served as the controls, while p62/*β*-actin levels were assessed and compared with the control group (∗*p* < 0.05 and ∗∗∗∗*p* < 0.0001 compared with the control group and #*p* < 0.05 compared to the Meth group). Figure S2. Meth treatment decreased the ability of pathological AD proteins' clearance in microglial cells. (a, b) The effect of Meth exposure on the co-localization of A*β*_1-42_ with Lysotracker in BV2 cells. Co-localization of LC3 and p62 was quantified and expressed as Pearson coefficient value (∗∗*p* < 0.01 compared with the control group). Scale bar: 20 *μ*m. (c, d) Co-localization of LC3 and A*β*_1-42_ by an immunofluorescence analysis. Co-localization of LC3 and A*β*_1-42_ was quantified and expressed as Pearson coefficient value (∗∗*p* < 0.01 compared with the control group). Scale bar: 20 *μ*m.
