# Supplementary material for: Targeting Aβ and p-Tau Clearance in Methamphetamine-Induced Alzheimer's Disease-Like Pathology: Roles of Syntaxin 17 in Autophagic Degradation in Primary Hippocampal Neurons
Source: Oxid Med Cell Longev. 2022 May 18;2022:3344569. doi: 10.1155/2022/3344569 (PMC9132709; doi:10.1155/2022/3344569)
Supplement: Supplementary Materials — Figure S1. Rapa rescues autophagosome–lysosome fusion deficiency induced by Meth in neurons. (a, c) Hippocampal neurons were transiently transfected with an RFP-GFP tandem fluorescent-tagged LC3 (RFP-GFP-LC3) and indicated with rapa and Meth (900 μm) for 24 h. The formation of autophagosome (RFP-positive; GFP-positive) and autophagolysosome (RFP-positive; GFP-negative) were detected and quantified by ImageJ (∗∗∗∗p < 0.0001 compared with the control group, ###p < 0.001 compared to the Meth group). Scale bars: 5 μm. (b, d) Western blot analysis of the effect of Meth (900 μm) and Rapa (25 μm) on LC3 and p62 in primary hippocampal neurons. LC3-II/LC3-I protein expression levels were assessed and served as the controls, while p62/β-actin levels were assessed and compared with the control group (∗p < 0.05 and ∗∗∗∗p < 0.0001 compared with the control group and #p < 0.05 compared to the Meth group). Figure S2. Meth treatment decreased the ability of pathological AD proteins' clearance in microglial cells. (a, b) The effect of Meth exposure on the co-localization of Aβ1-42 with Lysotracker in BV2 cells. Co-localization of LC3 and p62 was quantified and expressed as Pearson coefficient value (∗∗p < 0.01 compared with the control group). Scale bar: 20 μm. (c, d) Co-localization of LC3 and Aβ1-42 by an immunofluorescence analysis. Co-localization of LC3 and Aβ1-42 was quantified and expressed as Pearson coefficient value (∗∗p < 0.01 compared with the control group). Scale bar: 20 μm. [file 3344569.f1.docx]

**Supplementary materials**

**
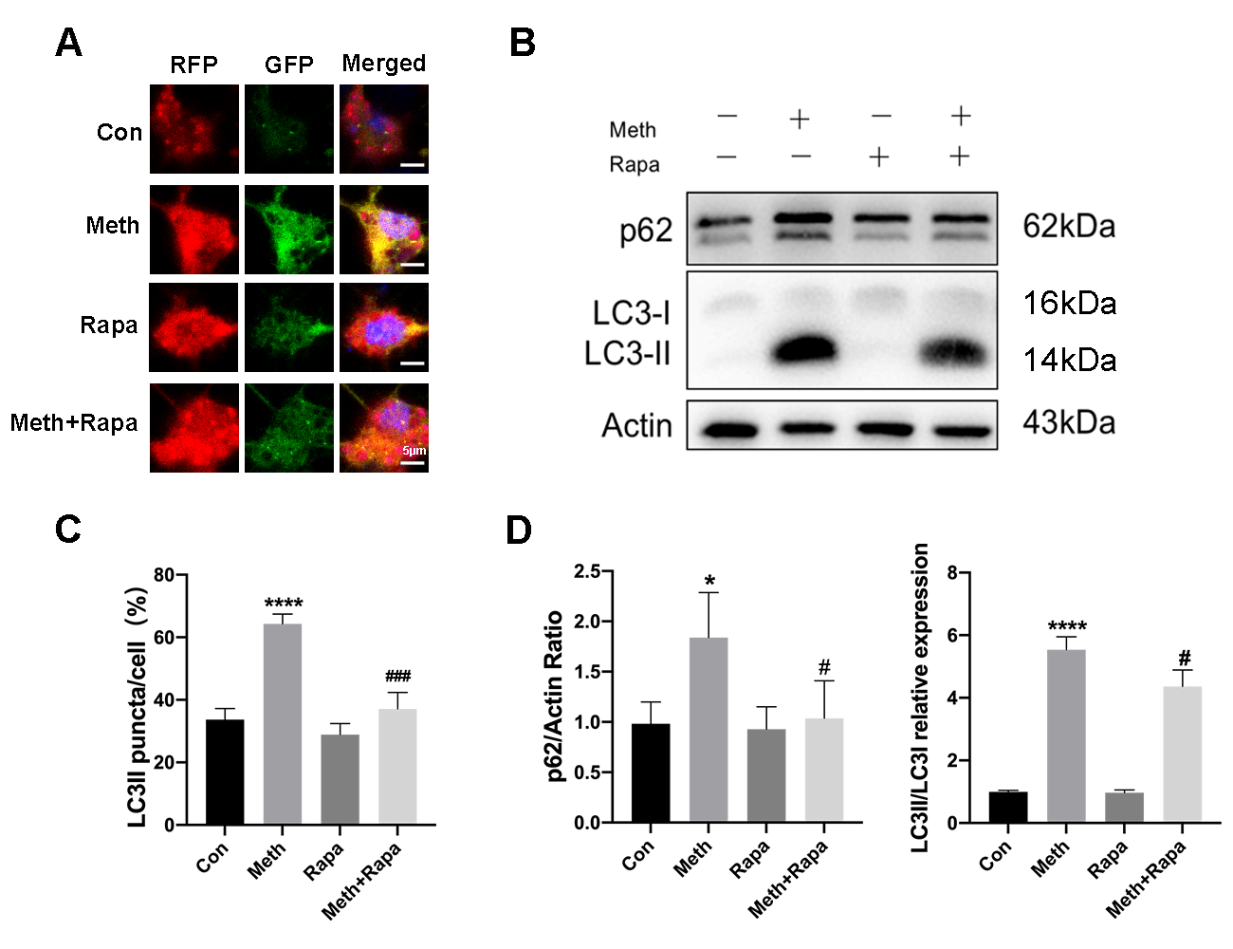
**

Figure S1

Rapa rescues autophagosome–lysosome fusion deficiency induced by Meth in neurons. (A,C）Hippocampal neurons were transiently transfected with an RFP-GFP tandem ﬂuorescent-tagged LC3 (RFP-GFP-LC3) and indicated with rapa and Meth (900 μm) for 24 h. The formation of autophagosome (RFP-positive; GFP-positive) and autophagolysosome (RFP-positive; GFP-negative) were detected and quantified by ImageJ (****p < 0.0001 compared with the control group, ^###^p < 0.001 compared to the Meth group). Scale bars: 5 μm. (B, D) Western blot analysis of the effect of Meth (900 μm) and Rapa (25 μm) on LC3 and p62 in primary hippocampal neurons. LC3-II/LC3-I protein expression levels were assessed and served as the controls, while p62/β-actin levels were assessed and compared with the control group (*p < 0.05, ****p < 0.0001 compared with the control group, ^#^p < 0.05 compared to the Meth group).


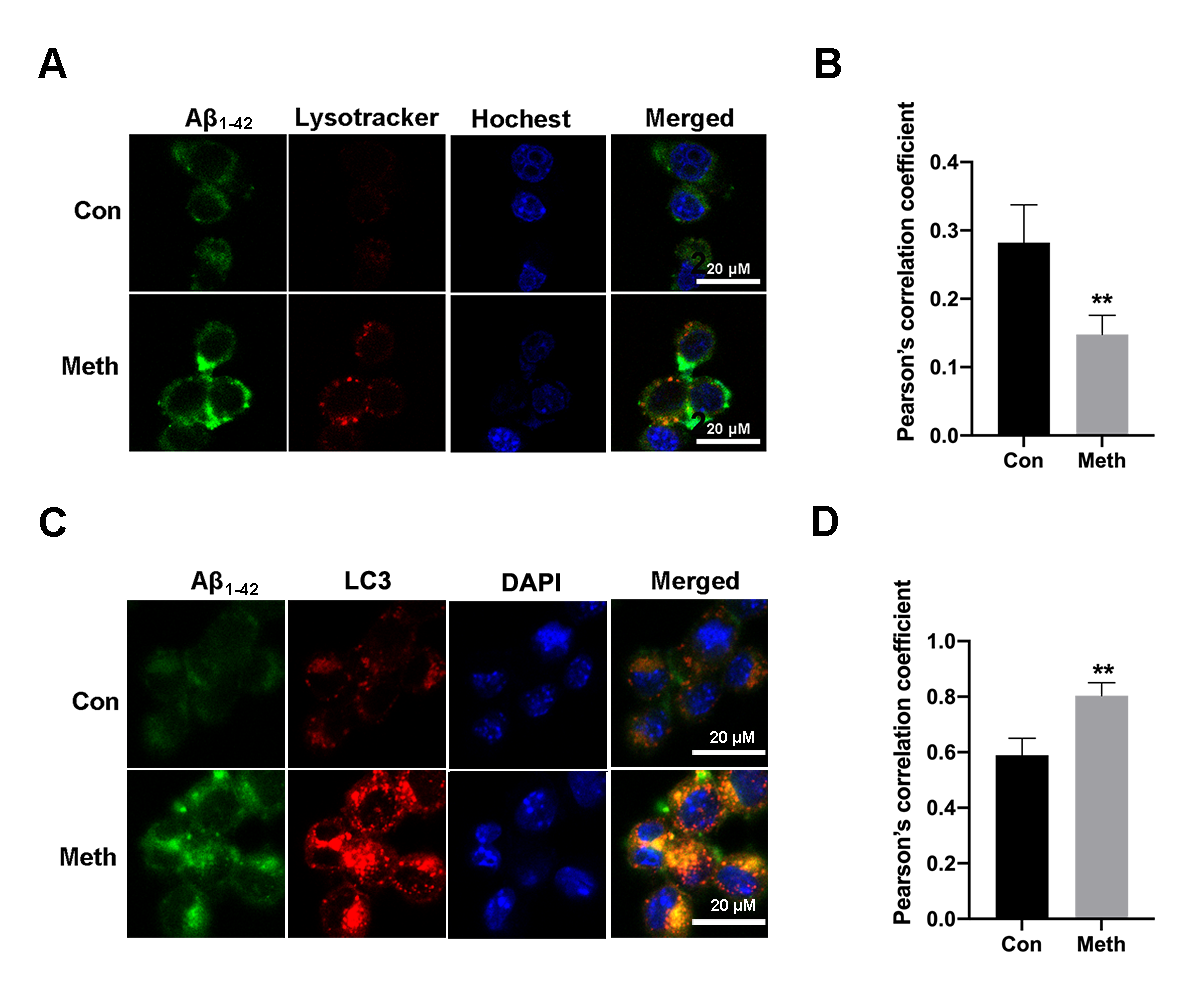


Figure S2

Meth treatment decreased the ability of pathological AD proteins’ clearance in microglial cells.

(A, B) The effect of Meth exposure on the colocalization of Aβ_1-42_ with Lysotracker in BV2 cells. Colocalization of LC3 and p62 was quantified and expressed as Pearson coefficient value (**p <0.01 compared with the control group). Scale bar: 20 μm. (C, D) Colocalization of LC3 and Aβ_1-42_ by an immunofluorescence analysis. Colocalization of LC3 and Aβ_1-42_ was quantified and expressed as Pearson coefficient value (**p <0.01 compared with the control group). Scale bar: 20μm.
